# Safety and Efficacy of Stereotactic Aspiration with Fibrinolysis for Supratentorial Spontaneous Intracerebral Hemorrhages: A Single-Center Experience

**DOI:** 10.3390/jcm14113636

**Published:** 2025-05-22

**Authors:** Chia-Ning Chang, Chiung-Chyi Shen, Meng-Yin Yang, Wen-Yu Cheng, Chih-Ming Lai

**Affiliations:** 1 Department of Neurosurgery, Neurological Institute, Taichung Veterans General Hospital, Taichung 407219, Taiwan; ning199001@gmail.com (C.-N.C.); yangmy04@gmail.com (M.-Y.Y.); 2 Department of Minimally Invasive Skull Base Neurosurgery, Neurological Institute, Taichung Veterans General Hospital, Taichung 407219, Taiwan; ccshen@vghtc.gov.tw (C.-C.S.); wycheng07@yahoo.com.tw (W.-Y.C.); 3 Department of Physical Therapy, Hung Kuang University, Taichung 433304, Taiwan; 4 Basic Medical Education, Central Taiwan University of Science and Technology, Taichung 40601, Taiwan; 5 Department of Post-Baccalaureate Medicine, College of Medicine, National Chung Hsing University, Taichung 402202, Taiwan; 6 Basic Medical Education Center, Central Taiwan University of Science and Technology, Taichung 40601, Taiwan; 7 Institute of Biomedical Sciences, National Chung Hsing University, Taichung 402202, Taiwan

**Keywords:** intracranial hemorrhage, stereotactic aspiration, fibrinolysis, modified Rankin scale

## Abstract

**Background/Objectives**: In recent years, stereotactic aspiration followed by fibrinolysis has been accepted as being a less invasive and more effective treatment for spontaneous intracerebral hemorrhage (ICH). The aim of this study was to evaluate the safety and clinical outcomes of frameless stereotactic aspiration and fibrinolysis using urokinase in a single medical center. **Methods**: This study included 123 patients with spontaneous supratentorial ICH who were treated with stereotactic aspiration and subsequent fibrinolysis using urokinase. Their clinical status, radiological images, and functional outcomes were assessed. **Results**: Unfavorable outcomes at discharge were associated with each patient’s preoperative Glascow Coma Score, as well as their initial and residual volumes of hematoma. Low mortality and minimal complications of rebleeding were also recorded. **Conclusions**: The results revealed that stereotactic aspiration and subsequent fibrinolysis with urokinase appeared to be a safe and feasible treatment modality for treating ICH. Further studies are still needed in order to better assess the optimal therapeutic window, thrombolytic dosage, long-term evaluation, and controlled comparisons of mortality, as well as disability outcomes in treated and untreated patients.

## 1. Introduction

Spontaneous intracerebral hemorrhage (ICH) is the stroke subtype with the greatest mortality rate, accounting for approximately 35% of all strokes occurring in Taiwanese patients [1], resulting in a 30–50% mortality rate at 30 days [2]. The risk of ICH is approximately twice as high in Asian populations as in White populations [3]. Furthermore, more than 30% of survivors experience different degrees of disability [4]. In recent years, the role of minimally invasive surgery (MIS) in the treatment of ICH has gained importance and proven to be an effective method for ICH management. Many studies have tested the safety and efficacy of stereotactic clot aspiration, combining it with the use of fibrinolysis in patients with cerebral hemorrhage [5,6,7,8,9,10,11,12,13,14,15,16,17]. Accordingly, the present study aimed to investigate the effect of stereotactic aspiration and subsequent fibrinolytic therapy for the treatment of spontaneous ICH.

## 2. Materials and Methods

### 2.1. Study Design

This was a retrospective study conducted during the period of July 2012 to August 2022, involving 123 patients with spontaneous ICH who were treated with stereotactic aspiration and subsequent fibrinolysis using urokinase in our tertiary hospital. The study inclusion criteria were lobar ICHs and supratentorial ICHs located in the basal ganglia and thalamus that were treated acutely upon arriving at our hospital. The study exclusion criteria were patients at an age of less than 18 years; secondary ICH due to trauma or tumor; underlying vascular abnormalities (arteriovenous malformation or cerebral aneurysm); infratentorial or brainstem ICH extension; and signs of transtentorial herniation.

### 2.2. Measurements of Radiological Parameters

A baseline computed tomography (CT) scan was obtained in all patients through axial images at a 5 mm slice thickness, with the dimensions of the hematoma subsequently assessed. The volume of the ICH in milliliters was estimated on the basis of the approximate ellipse volume using the A*B*C/2 formula, where A is the largest diameter of the hematoma on the axial CT cuts in centimeters, B is the diameter of the hematoma perpendicular to A on the same cut, and C is the approximate number of CT slices with hemorrhage [5,16]. Intravenous contrast was administered to assess for any enhancement which would appear suspicious for an underlying structural lesion.

### 2.3. Surgical Procedure

Stereotactic catheter aspiration was conducted using a frameless stereotactic navigation system (BrainLab AG, Munich, Germany) or syngo iGuide needle guidance (Siemens Healthineers, Forchheim, Germany) [18]. All surgeries were conducted by a well-trained surgical team.

**Figure 1:**
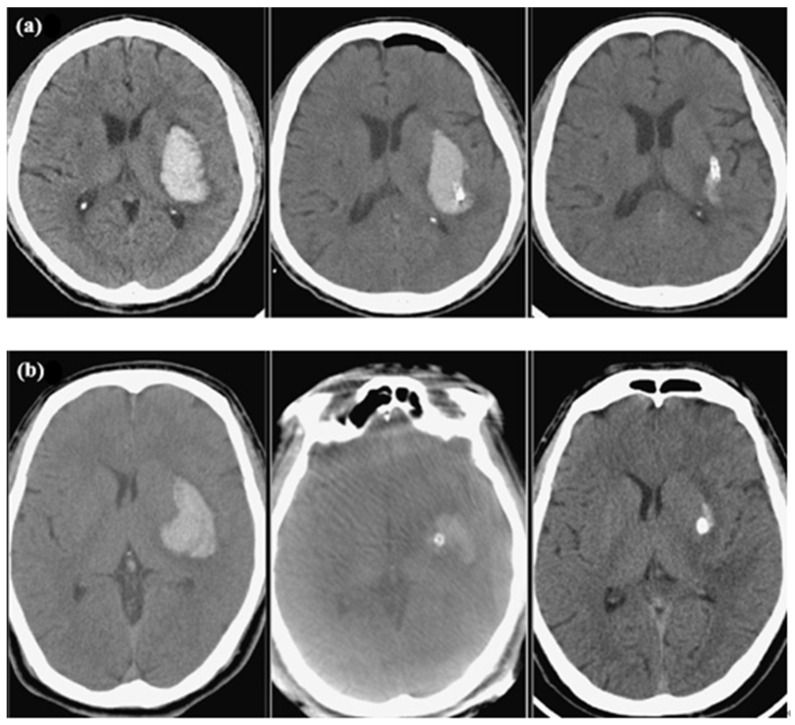
CT scans showing examples of left ganglionic ICH patients at baseline presentation (**left**), after aspiration with catheter placement (**middle**), and in a post-fibrinolytic state (**right**). All scans demonstrate the largest size of the hematoma on axial images. The procedure was conducted using either Brainlab navigation (**a**) or syngo iGuide needle guidance (**b**).

Briefly, a thin-section CT scan (1 mm) was performed prior to surgery for the BrainLab navigation data construction, while a flat panel detector CT image was obtained in the angiography suite for syngo workstation reconstruction. The entry point and trajectory were designed along the long axis of the hematoma. At this entry point, a 2–3 cm incision was made and a burr hole was then fashioned with a high-speed electric drill, with the dura opening sharply. An external ventricular catheter (Medtronic, O.D. 4.9 mm) with a rigid cannula was placed into the hematoma via navigated guidance through the optimal trajectory and depth. Our catheter tip was targeted near the posterior end of the hematoma which was gently aspirated using a 10 mL volume syringe until resistance was reached. Saline was used to wash the hematoma cavity until no further blood clots could be aspirated. The intraclot catheter was tunneled subcutaneously and then sutured in place. An additional CT scan was performed after surgery to determine the proper location of the catheter. Subsequently, fibrinolytic therapy was performed, involving 6000 units of urokinase (diluted with 3 cc of preservative-free 0.9% saline units) being injected into the hematoma cavity and left for 30–60 min with the catheter clamped and re-opened to allow for drainage of the lysed clot without a pressure gradient. This procedure was repeated 2–4 times per day for 2–6 days depending on the amount of fluid drainage and the results from repeated CT scans (Figure 1). The catheter was removed at the bedside through the use of a sterile technique, and a single suture was placed at its exit site and covered with an occlusive dressing.

### 2.4. Patient Care

All patients were admitted to the neurosurgical intensive care unit until they were evaluated as being stable enough for the general ward. We followed the current guidelines for the management of spontaneous ICH taken from the American Heart Association and American Stroke Association with standard care for monitoring patients’ blood pressure, airways, pharmacological treatment, and physiotherapy.

### 2.5. Data Collection

The demographic and clinical data of the patients (age, gender, history of hypertension (HTN) and diabetes mellites (DM), Glasgow Coma Scale (GCS), modified Rankin Scale (mRS), ICH score, time to evacuation (from symptom onset), permanent shunt placement, total dose of urokinase, etc.) were all reviewed from the inpatient medical record system. Radiological data were assessed using the CT images taken from the radiology imaging system, including the presence of intraventricular hemorrhage (IVH) and midline shift, and the location and volume of the hematoma. Depending on the localization, the hematomas were classified into either lobar or deep-seated (basal ganglia, thalamus) hematomas according to their origination. Evacuation percent was calculated as (post-fibrinolytic hematoma volume/initial hematoma volume) × 100%. Postoperative rebleeding was defined as hematoma volume expansion ≥ 5 mL between preoperative and post-fibrinolytic CT scans.

### 2.6. Clinical Outcome Assessment

Functional outcomes assessed by the mRS score were followed up at discharge. mRS scores ranging from 0 to 3 were considered favorable outcomes, while those ranging from 4 to 6 were considered unfavorable outcomes. In addition, in-hospital mortality, hospital length of stay, and complications such as systemic infection (sepsis) were also reviewed.

### 2.7. Statistical Analysis

By using Fisher’s exact test, the chi-squared test, and the Mann–Whitney U test, we evaluated the association of discharge outcome (favorable vs. unfavorable) and evacuation percentage with patients’ age (in years), preoperative GCS score, initial ICH volume (in mL), presence of IVH and midline shift on the admission CT scan, time to evacuation, residual clot volume, GCS at discharge, total dose of urokinase administration, and ICH score.

Logistic regression analysis was performed to analyze the prognostic impact of the following parameters on discharge mRS: gender, age, history of DM/HTN, preoperative clinical status, hematoma volume before and after treatment, ICH score, location of hematoma, ventricular extension, and time to evacuation. For that purpose, mRS was dichotomized into favorable (mRS ≤ 3) or unfavorable outcome (mRS > 3). Statistical significance was set at *p* < 0.05.

## 3. Results

A total of 123 patients diagnosed with spontaneous ICH who were treated with stereotactic catheter aspiration were included in the analysis, consisting of 86 (69.9%) males and 37 (30.1%) females. The mean age of the cohort was 53.8 ± 15.6 (range, 22–90 years). Eighty-four (68.3%) patients had a history of HTN, while 16.3% had a history of DM. The mean GCS score on arrival was 11.3 ± 2.8 (range, 3–15). A total of 23 (18.7%) patients had a GCS score between 3 and 8; 54 (43.9%) had a score between 9 and 12; and 46 (37.4%) had a score between 13 and 15. A total of 58 (47.1%) patients underwent stereotactic catheter aspiration involving the frameless BrainLab navigation system, while 65 (52.8%) underwent the procedure with frameless iGuide navigation. The majority (83.7%) of hematomas were located in the basal ganglia, while twelve (9.8%) were located in the thalamus and eight (6.5%) within the lobes. Intraventricular extension was present in 31.7% of patients, and permanent shunt insertion was required in 8.9% of patients. Midline shift on the CT scan was recorded in 65% of patients. The mean initial ICH volume was 33.1 ±17.9 (range, 5–115 mL). The median ICH score was 2 (range, 0–4). The median time from ICH onset to the operation of hematoma aspiration was 10 h (IQR 6.3–20.3), with 95 (77.2%) patients having the surgery within 24 h of onset. Thrombolysis with urokinase was performed at a median dose of 6 (IQR, 4–7). The mean residual hematoma volume after treatment was reduced to 12.9 ± 13.5 mL, and the evacuation rate was 63.3 ± 24%. Only one patient experienced rebleeding after fibrinolysis, while three (2.4%) patients died during admission. The median discharge mRS was 4 (range, 1–6), with the majority (82.1%) of patients experiencing unfavorable outcomes (mRS 4–6). The detailed demographic and clinical data of the patients are listed in Table 1.

Factors associated with unfavorable outcomes at discharge in univariate analysis are demonstrated in Table 2. There were no statistically significant differences in age, gender, history of HTN/DM, location/side of ICH, total dose of urokinase, and permanent shunt requirement between the two groups (mRS 1–3 vs. mRS 4–6). Preoperative GCS score, initial hematoma volume, presence of IVH, residual clot volume, and length of stay all presented significant statistical differences between the two groups.

The patients in the mRS (4–6) group had a lower preoperative and discharge GCS score when compared to the mRS (0–3) group (*p* < 0.01). Among patients with an initial GCS score in the range of 3–8, no patient had a favorable outcome; among patients with an initial GCS score in the range of 9–12, 6 (27.3%) had a favorable outcome; and among patients with a GCS score in the range of 13–15, 16 (72.7%) patients experienced a favorable outcome. A larger initial volume of hematoma was shown to be a significant predictor of unfavorable outcomes (*p* < 0.01), as well as a larger residual hematoma after fibrinolysis (*p* < 0.01).

No significant differences between the favorable and unfavorable groups were observed in complications, including postoperative rebleeding and sepsis, total dose of urokinase, time to evacuation, and percentage of residual hematoma volume when compared to initial volume.

Logistic regression analysis identified preoperative GCS score (OR 0.67, *p* < 0.01; dichotomized < 12: OR 5.38, *p* < 0.01), initial hematoma volume (OR 1.08, *p* < 0.01; dichotomized > 24 mL: OR 9.81, *p* < 0.01), residual hematoma volume after fibrinolysis (OR 1.12, *p* < 0.01; dichotomized > 4 mL: OR 5.5, *p* < 0.01), and length of stay (OR 1.12, *p* < 0.01; dichotomized > 20 days: OR 5.87, *p* < 0.01) as risk factors for an unfavorable discharge clinical outcome (mRS > 3) (Table 3a,b).

Greater ICH volumes were significantly associated with IVH presence (*p* < 0.01) and midline shift (*p* < 0.01). Furthermore, those patients who presented with IVH had a 12-fold increase in unfavorable outcomes (OR 12.67, *p* < 0.05), with the presence of midline shift also being significantly associated with unfavorable outcomes (OR 5.59, *p* < 0.01).

Subgroup analysis showed no significant correlation between time to evacuation and hematoma evacuation percentage (Table 4a). There was no statistically significant correlation seen between hematoma evacuation percentage and total dose of urokinase administration (Table 5).

In addition, as aspiration was performed in a delayed fashion (>48 h), the mean preoperative GCS score was statistically significantly higher (*p* < 0.05) while the mean initial volume was significantly lower (*p* < 0.01) with a lower residual clot volume (Table 4b).

## 4. Discussion

Our data show that both preoperative clinical status and post-treatment variables have significant effects on short-term functional outcomes, including a preoperative GCS score < 12, an initial hematoma volume > 24 mL, and a residual hematoma volume after fibrinolysis > 4 mL. Similar results have been demonstrated in several previous studies.

Thiex et al. reported that the risk factors for an unfavorable clinical outcome (Glasgow outcome score (GOS) ≤ 3) at discharge were a preoperative hematoma volume > 50 mL and a remaining hematoma volume after lysis > 10 mL [6]. Bernotas et al. showed that patients with unfavorable outcomes (GOS ≤ 3) at discharge had lower GCS scores at admission [7]. It was also noticed that there was a correlation between GCS score on arrival and ICH volume, making for strong predictors of short-term outcomes [7,19].

The burden of an intracerebral hematoma initiates a cascade of secondary events through blood products and inflammatory responses, leading to intracranial hypertension, brain edema, neuronal damage, and disruption of the blood–brain barrier [2,16,20]. The degree of secondary brain injury is related to the exposure time (of the brain to the hematoma) and the volume of the hematoma [11]. Therefore, early intervention and as much hematoma evacuation as can be achieved are believed to alleviate the injury, corresponding to several studies’ results [12]. However, the impact of the timing of surgery for ICH patients on functional outcomes remains controversial.

Fang et al. reported that patients with early hematoma evacuation (<48 h after ICH onset) were less likely to experience poor outcomes (mRS > 3) both 3 months and 1 year after discharge [12]. A meta-analysis demonstrated that MIS evacuation within 24 h was more likely to achieve functional independence [21]. The ENRICH trial showed MIS evacuation of hematomas within 24 h in patients with lobar ICH, with improved functional outcomes at 180 days, but not in those with basal ganglia hemorrhages [22]. The effect of surgery may differ by the degree and time frame of ICH, and also the hemorrhage location.

Contrarily, our study showed that the time from ictus to surgery did not significantly influence short-term outcomes. However, subgroup analysis showed that favorable outcomes (mRS ≤ 3) were significantly more frequent in the group with a delayed aspiration of more than 48 hrs. This is probably associated with delayed aspiration usually being performed in neurologically stable patients with a lower preoperative hematoma volume in our practices. The usual presence of both a large hematoma volume and a deteriorated level of consciousness in these patients who received early surgery may outweigh the beneficial effect of timing for surgery.

Some studies have raised concerns that very early surgery may be dangerous due to an increased risk of rebleeding, which usually occurs within 4 h of onset [8]. Several authors have suggested avoiding aspiration and thrombolysis in the initial 6 to 24 h after ICH onset [16].

In the present study, there was only one patient (1/123, 0.8%) who suffered from post-fibrinolytic rebleeding, which is a lower percentage than that seen in recent studies (4.9–22.2%) [12]. Our study showed that it is both feasible and safe for surgery to occur even earlier than 6 h post ictus. Avoiding violent aspiration through the use of urokinase to liquefy the hematoma may be vital in minimizing the incidence of rebleeding [8].

Of note, MIS evacuation up to 72 h has even shown efficacy over conventional therapy [21]. Minimum volume evacuation thresholds, as well as a broad therapeutic time window, in order to achieve the greatest probability of functional benefit, were also noted in the MISTIE III and STICH I and II studies [23]. A trend toward a higher probability of good functional outcomes (mRS 0–3) at 180 days was found between 90 and 120 h post ictus in the MISTIE III cohort and 48 and 72 h in the STICH I and II cohorts [23]. There still remains great interest in what is the optimal timing from ictus to surgical intervention, so further research is certainly warranted. We suggest a policy of relatively aggressive care (including this MIS procedure) for patients who are healthy without advance directives before a broad time window.

In addition, patients with a residual hematoma of less than 15 mL were prone to achieving favorable outcomes (mRS ≤ 3) both at their discharge and 6-month follow-up [14,17]. There was also a significant improvement in mRS at one year for those patients who achieved a hematoma volume reduction to below 15 mL in the MISTIE III trial [24].

Sirh et al. reported that the residual volume ratio of the CT scan immediately after aspiration was significantly lower with delayed aspiration (>7 days). However, a similar amount of residual hematoma was left after drainage with or without the use of a thrombolytic agent. No impact of timing on neurologic outcomes either at 1 month or 6 months postoperatively was shown, but the volume of the final hematoma after drainage had a significant correlation with a favorable outcome 6 months postoperatively [11].

In our study, this kind of MIS intervention seemed to be effective in hematoma evacuation in most of the patients with deep basal ganglia ICH, with emphasis on achieving the desired clot reduction.

IVH was found to be present in approximately 32% of ICH patients in our findings. The occurrence of IVH is also consistently associated with worse outcomes, as determined in previous studies [14,25,26]. Hallevi et al. identified IVH in 45% of patients with spontaneous ICH and found that a larger ICH volume was significantly correlated with the presence of IVH. Those who presented with IVH had a 2-fold increase in poor outcomes (discharge mRS > 3) [27]. Early therapeutic intervention remains critical for minimizing the risk of mortality and IVH-induced morbidity, including the development of hydrocephalus, decreased consciousness, and inflammation [27].

However, due to the retrospective nature of our study and the transfer program of the patients, which sends them to a local hospital for further rehabilitation, it has become difficult to evaluate the long-term outcomes of these patients. In the present study, though the mRS scores at discharge seemed poor, the proportion of favorable outcomes in long-term follow-up may increase at the conclusion of rehabilitation, as evidenced by the result of a 4-fold increase in functional independence seen between Day 30 and Day 365 from the MISTIE III study [28].

It is noteworthy that the mortality rate of 3% in our study compares favorably with recent studies [6,9,12,17,20]. We have found that the minimally invasive surgery involving stereotactic aspiration and subsequent fibrinolysis has its own promising role in ICH evacuation, particularly in deep-seated hematoma patients under a relatively broad inclusion criterion, although the probability of favorable functional outcomes is low.

## 5. Limitations

The major limitations of the present study include its retrospective, single-center analysis, its relatively small sample size, its limited follow-up period, and the outcome assessment being restricted to the patients’ mRS scores. Other important parameters, including cognitive disability and language assessment, have not been assessed. A longer follow-up period may provide a more comprehensive view of the outcome evaluation, since the recovery of patients with ICH is long. Lastly, there was no medical control group involved to investigate the benefits of intervention and potential confounding factors. A large prospective study is still required in order to determine the relationship between the timing of surgery, urokinase dosage, degree of evacuation of ICH aspiration, and clinical prognosis.

## 6. Conclusions

This study has shown that frameless stereotactic aspiration of spontaneous ICH followed by fibrinolytic therapy is an effective and safe procedure with a lower rebleeding rate and mortality. Short-term functional outcomes were associated with the preoperative clinical condition and significant hematoma resolution. Further larger randomized trials are still required in order to better investigate and validate the therapeutic efficacy, optimal timing, and long-term outcome prediction of this type of treatment.

## Figures and Tables

**Table 1:** Demographic and baseline characteristics of patients.

	Total (*n* = 123)
Age (years), mean ± SD	53.8 ± 15.6
Gender, *n* (%)	
Female	37 (30.1%)
Male	86 (69.9%)
Preoperative GCS score, mean ± SD	11.3 ± 2.8
Preoperative GCS score, *n* (%)	
GCS score 13–15	46 (37.4%)
GCS score 9–12	54 (43.9%)
GCS score 3–8	23 (18.7%)
Hypertension, *n* (%)	84 (68.3%)
Diabetes, *n* (%)	20 (16.3%)
Location, *n* (%)	
Basal ganglia	103 (83.7%)
Thalamus	12 (9.8%)
Lobar	8 (6.5%)
Side, *n* (%)	
Right	53 (43.1%)
Left	70 (56.9%)
IVH, *n* (%)	39 (31.7%)
Midline shift, *n* (%)	80 (65%)
VP shunt, *n* (%)	11 (8.9%)
Preoperative volume (mL), mean ± SD	33.1 ± 17.9
Preoperative volume (mL), *n* (%)	
<30 mL	61 (49.6%)
30–50 mL	42 (34.1%)
>50 mL	20 (16.3%)
Residual clot volume (mL), mean ± SD	12.9 ± 13.5
Residual ≤ 15 mL, *n* (%)	81 (65.9%)
Residual percentage (%), mean ± SD	36.7 ± 24
Total dose of urokinase, mean ± SD	5.5 ± 3
Sepsis, *n* (%)	11 (8.9%)
Discharge GCS score, mean ± SD	13.3 ± 2.1
Discharge GCS score, *n* (%)	
GCS score 13–15	86 (71.7%)
GCS score 9–12	28 (23.3%)
GCS score 3–8	6 (5%)
Time to evacuation (hrs), mean ± SD	25.8 ± 43.8
Time to evacuation (hrs), *n* (%)	
≤6 h	28 (22.8%)
6–12 h	43 (34.9%)
12–24 h	24 (19.5%)
>24 h	28 (22.8%)
Evacuation percentage (%), mean ± SD	63.3 ± 24
Evacuation percentage (%), *n* (%)	
>75% reduction	41 (33.3%)
51–75% reduction	48 (39%)
26–50% reduction	24 (19.5%)
≤25% reduction	10 (8.2%)
Discharge mRS, mean ± SD	4.4 ± 1.1
Discharge mRS, *n* (%)	
mRS 1–3	22 (17.9%)
mRS 4–6	101 (82.1%)
ICH score, mean ± SD	1.5 ± 1.1
ICH score, *n* (%)	
ICH score 0–2	99 (80.5%)
ICH score 3–4	24 (19.5%)
Postoperative rebleeding, *n* (%)	1 (0.8%)
Mortality, *n* (%)	3 (2.4%)
Follow-up time (days), mean ± SD	25.5 ± 11.6

mRS, modified Rankin Scale; SD, standard deviation; GCS, Glasgow Coma Scale; VP shunt, ventriculoperitoneal shunt; ICH, intracerebral hemorrhage; IVH, intraventricular hemorrhage.

**Table 2:** Comparison between patients with favorable outcomes and patients with unfavorable outcomes regarding clinical data, radiological results, and complications at discharge.

	Discharge mRS		
Variable	mRS 1–3 (*n* = 22)	mRS 4–6 (*n* = 101)	*p* Value
Age (years), mean ± SD	48.3 ± 15.4	55 ± 15.4	0.061
Gender, *n* (%)			0.845
Female	7 (31.8%)	30 (29.7%)	
Male	15 (68.2%)	71 (70.3%)	
**Preoperative GCS score, mean ± SD**	**13.2 ± 2**	**10.9 ± 2.8**	<0.001 **
Preoperative GCS score, *n* (%)			<0.001 **
GCS score 13–15	16 (72.7%)	30 (29.7%)	
GCS score 9–12	6 (27.3%)	48 (47.5%)	
GCS score 3–8	0 (0%)	23 (22.8%)	
Hypertension, *n* (%)	17 (77.3%)	67 (66.3%)	0.318
Diabetes, *n* (%)	2 (9.1%)	18 (17.8%)	0.524
Location, *n* (%)			0.082
Basal ganglia	19 (86.4%)	84 (83.1%)	
Thalamus	0 (0%)	12 (11.9%)	
Lobar	3 (13.6%)	5 (5%)	
Side, *n* (%)			0.482
Right	8 (36.4%)	45 (44.6%)	
Left	14 (63.6%)	56 (55.4%)	
**IVH, *n* (%)**	**1 (4.5%)**	**38 (37.6%)**	0.003 **
**Midline shift, *n* (%)**	**7 (31.8%)**	**73 (72.3%)**	<0.001 **
VP shunt, *n* (%)	0 (0%)	11 (10.9%)	0.211
**Preoperative volume (mL), mean ± SD**	**21 ± 11.6**	**35.8 ± 18**	<0.001 **
Preoperative volume (mL), *n* (%)			0.015 *
<30 mL	17 (77.3%)	44 (43.6%)	
30–50 mL	4 (18.2%)	38 (37.6%)	
>50 mL	1 (4.5%)	19 (18.8%)	
**Residual clot volume (mL), mean ± SD**	**6.3 ± 6.4**	**14.4 ± 14.2**	0.001 **
Residual ≤ 15 mL, *n* (%)	20 (90.9%)	61 (60.4%)	0.006 **
Residual percentage (%), mean ± SD	30.3 ± 27.8	38.1 ± 23	0.075
Total dose of urokinase, mean ± SD	4.9 ± 2.3	5.7 ± 3.1	0.347
Sepsis, *n* (%)	1 (4.5%)	10 (9.9%)	0.687
**Discharge GCS score, mean ± SD**	**14.9 ± 0.4**	**13 ± 2.2**	<0.001 **
Discharge GCS score, *n* (%)			0.003 **
GCS score 13–15	22 (100%)	64 (65.3%)	
GCS score 9–12	0 (0%)	28 (28.6%)	
GCS score 3–8	0 (0%)	6 (6.1%)	
Time to evacuation (hrs), mean ± SD	46.5 ± 62.9	21.2 ± 37.4	0.050
Time to evacuation (hrs), *n* (%)			0.009 **
≤48 h	15 (68.2%)	92 (91.1%)	
>48 h	7 (31.8%)	9 (8.9%)	
Evacuation percentage (%), mean ± SD	69.7 ± 27.8	61.9 ± 23	0.075
**ICH score, mean ± SD**	**0.5 ± 0.8**	**1.8 ± 1.1**	<0.001 **
ICH score, *n* (%)			0.072
ICH score 0–2	21 (95.5%)	78 (77.2%)	
ICH score 3–4	1 (4.5%)	23 (22.8%)	
Postoperative rebleeding, *n* (%)	0 (0%)	1 (1%)	1.000
Mortality, *n* (%)	0 (0%)	3 (3%)	1.000
**Follow-up time (days), mean ± SD**	**17.7 ± 8.3**	**27.2 ± 11.6**	<0.001 **

Mann–Whitney test. Chi-squared test. Fisher’s exact test. * *p* < 0.05. ** *p* < 0.01. mRS, modified Rankin Scale; SD, standard deviation; GCS, Glasgow Coma Scale; VP shunt, ventriculoperitoneal shunt; ICH, intracerebral hemorrhage; IVH, intraventricular hemorrhage.

**Table 3:** (a) Risk factors of unfavorable mRS were established using a logistic regression model. (b) Cutoff value for preoperative GCS score, initial and residual volumes of hematoma, follow-up time between patients with unfavorable mRS, and ROC curve.

**(a)**			
**Components of Regression Model**	**Odds Ratio**	**(95% CI)**	***p* Value**
Age	1.03	(1.00–1.07)	0.070
Gender			
Female	Reference		
Male	1.10	(0.41–2.98)	0.845
**Preoperative GCS score**	**0.67**	**(0.53**–**0.85)**	0.001 **
Hypertension	0.58	(0.20–1.71)	0.322
Diabetes	2.17	(0.46–10.12)	0.325
Side			
Right	Reference		
Left	0.71	(0.27–1.84)	0.483
IVH	12.67	(1.64–98.01)	0.015 *
Midline shift	5.59	(2.06–15.15)	0.001 **
**Preoperative volume (mL)**	**1.08**	**(1.03**–**1.14)**	0.001 **
<30 mL	Reference		
30–50 mL	3.67	(1.14–11.86)	0.030 *
>50 mL	7.34	(0.91–59.19)	0.061
**Residual clot volume (mL)**	**1.12**	**(1.04**–**1.21)**	0.004 **
Residual ≤ 15 mL	0.15	(0.03–0.69)	0.014 *
Residual percentage (%)	1.02	(0.99–1.04)	0.171
Total dose of urokinase	1.09	(0.93–1.28)	0.284
Sepsis	2.31	(0.28–19.03)	0.437
Discharge GCS score	0.14	(0.04–0.47)	0.002 **
Time to evacuation (h)	0.99	(0.98–1.00)	0.029 *
Evacuation percentage (%)	0.99	(0.96–1.01)	0.171
ICH score	3.83	(2.01–7.31)	<0.001 **
**Follow-up time (days)**	**1.12**	**(1.05**–**1.19)**	0.001 **
**(b)**			
	**Discharge mRS**		***p* Value**
	**mRS 1–3 (*n* = 22)**	**mRS4–6 (*n* = 101)**	
Preoperative GCS score < 12, *n* (%)	4 (18.2%)	55 (54.5%)	0.002 **
Preoperative volume (mL) > 24, *n* (%)	5 (22.7%)	75 (74.3%)	<0.001 **
Residual clot volume (mL) > 4, *n* (%)	9 (40.9%)	80 (79.2%)	<0.001 **
Follow-up time (days) > 20, *n* (%)	7 (31.8%)	74 (73.3%)	<0.001 **
*mRS*, modified Rankin Scale; *GCS*, Glasgow Coma Scale			
	**AUC**	**(95% CI)**	***p* value**
Preoperative GCS score	0.75	(0.65–0.85)	<0.001 **
Preoperative volume (mL)	0.78	(0.67–0.89)	<0.001 **
Residual clot volume (mL)	0.73	(0.62–0.85)	0.001 **
Follow-up time (days)	0.75	(0.64–0.87)	<0.001 **

* *p* < 0.05, ** *p* < 0.01. AUC, area under the curve.

**Table 4:** (a) Characteristics of patient group according to timing of hematoma evacuation and associated factors. (b) Subgroup analysis of delayed timing of hematoma evacuation and associated factors.

**(a)**	**Time to Evacuation (h)**				***p* Value**
	**≤6 h (*n* = 28)**	**6–12 h (*n* = 43)**	**12–24 h (*n* = 24)**	**>24 h (*n* = 28)**	
Preoperative GCS score, mean ± SD	9.9 ± 2.6	11.2 ± 2.9	11.5 ± 2.7	12.8 ± 2.3	0.001 **
Preoperative volume (mL), mean ± SD	36.9 ± 15.5	36.5 ± 21	31.1 ± 15.5	25.8 ± 15.1	0.035 *
Residual clot volume (mL), mean ± SD	13.5 ± 10.5	15.4 ± 18.3	13.3 ± 10.5	8.3 ± 7.8	0.073
Total dose of urokinase, mean ± SD	5.9 ± 2.7	5.3 ± 3.1	6 ± 3.3	5 ± 2.9	0.528
Evacuation percentage (%), mean ± SD	63.4 ± 22.1	62.7 ± 21.8	58.5 ± 25	68.5 ± 28	0.286
Postoperative rebleeding, *n* (%)	0 (0%)	0 (0%)	0 (0%)	1 (3.6%)	0.650
**(b)**	**Time to Evacuation (h)**				***p* Value**
		**≤48 h (*n* = 107)**		**>48 h (*n* = 16)**	
Preoperative GCS score, mean ± SD		11.1 ± 2.8		12.8 ± 2.4	0.022 *
Preoperative volume (mL), mean ± SD		34.8 ± 17.8		21.8 ± 14.8	0.003 **
Residual clot volume (mL), mean ± SD		14.3 ± 13.8		3.9 ± 4.6	<0.001**
Evacuation percentage (%), mean ± SD		60.6 ± 23.5		81.5 ± 19.1	<0.001**

(a) Kruskal–Wallis test. Chi-squared test. Fisher’s exact test. * *p* < 0.05. ** *p* < 0.01. mRS, modified Rankin Scale; SD, standard deviation; GCS, Glasgow Coma Scale. (b) Mann–Whitney test. Chi-squared test. Fisher’s exact test. * *p* < 0.05. ** *p* < 0.01. SD, standard deviation; GCS, Glasgow Coma Scale.

**Table 5:** Characteristics of patient group according to timing of hematoma evacuation and associated factors.

	Evacuation Percentage, %				*p* Value
	>75% (n = 41)	51–75% (n = 48)	26–50% (n = 24)	≤25% (n = 10)	
Preoperative volume (mL), mean ± SD	29.3 ± 16.3	34.7 ± 16	34.4 ± 17.6	38.1 ± 30.4	0.364
Total dose of urokinase, mean ± SD	5.1 ± 2.2	6.1 ± 2.8	5 ± 3.9	6.1 ± 3.6	0.274
Time to evacuation (hrs), mean ± SD	47.3 ± 68.6	13.4 ± 12.3	16.6 ± 18.6	18.7 ± 15.8	0.118
Postoperative rebleeding, *n* (%)	0 (0%)	1 (2.1%)	0 (0%)	0 (0%)	1.000

Kruskal–Wallis test. Chi-squared test. Fisher’s exact test. mRS, modified Rankin Scale; SD, standard deviation; GCS, Glasgow Coma Scale.

## Data Availability

All data generated or analyzed during this study are included in this article. Further enquiries can be directed to the corresponding author.

## Abbreviations

The following abbreviations are used in this manuscript:

ICH	Intracerebral hemorrhage
MIS	Minimally invasive surgery
CT	Computed tomography
IVH	Intraventricular hemorrhage
mRS	Modified Rankin Scale
GCS	Glasgow Coma Scale
HTN	Hypertension
DM	Diabetes mellitus
